# Outer membrane vesicles derived from gut microbiota mediate tubulointerstitial inflammation: a potential new mechanism for diabetic kidney disease

**DOI:** 10.7150/thno.84650

**Published:** 2023-07-09

**Authors:** Pei Pei Chen, Jia Xiu Zhang, Xue Qi Li, Liang Li, Qin Yi Wu, Liang Liu, Gui Hua Wang, Xiong Zhong Ruan, Kun Ling Ma

**Affiliations:** 1 Institute of Nephrology, Zhongda Hospital, School of Medicine, Southeast University, Nanjing, 210009, China.; 2 People's Hospital Affiliated to Shandong First Medical University, Shandong, 271100, China.; 3 John Moorhead Research Laboratory, Department of Renal Medicine, University College London (UCL) Medical School, Royal Free Campus, London, NW3 2PF, UK.; 4 Department of Nephrology, the Second Affiliated Hospital, School of Medicine, Zhejiang University, Hangzhou, 310003, China.

**Keywords:** diabetic kidney disease, renal tubulointerstitial inflammation, gut microbiota, outer membrane vesicles, caspase-11 pathway

## Abstract

**Rationale:** Chronic tubulointerstitial inflammation is a common pathological process in diabetic kidney disease (DKD). However, its underlying mechanism is largely unknown. This study aims at investigating the role of gut microbiota-derived outer membrane vesicles (OMVs) in tubulointerstitial inflammation in DKD.

**Methods:** Gut microbiota in diabetes mellitus rats was manipulated by microbiota depletion and fecal microbiota transplantation to explore its role in tubulointerstitial inflammation. To check the direct effects of OMVs, fecal bacterial extracellular vesicles (fBEVs) were administrated to mice orally and HK-2 cells *in vitro*. For mechanistic investigations, HK-2 cells were treated with small interfering RNA against caspase-4 and fBEVs pre-neutralized by polymyxin B.

**Results:** By performing gut microbiota manipulation, it was confirmed that gut microbiota mediated tubulointerstitial inflammation in DKD. In diabetic rats, gut microbiota-derived OMVs were increased and were clearly detected in distant renal tubulointerstitium. Diabetic fBEVs directly administered by gavage translocated into tubular epithelial cells and induced tubulointerstitial inflammation and kidney injury. *In vitro*, OMVs were internalized through various endocytic pathways and triggered cellular inflammatory response. Mechanistically, it was revealed that OMVs-derived lipopolysaccharide induced tubular inflammation, which was mediated by the activation of the caspase-11 pathway.

**Conclusions:** Increased OMVs due to dysbiosis translocated through leaky gut barrier into distant tubulointerstitium and induced cellular inflammation and renal tubulointerstitial injury in DKD*.* These findings enrich the mechanism understanding of how gut microbiota and its releasing OMVs influence the development and progression of kidney disease.

## Introduction

Diabetic kidney disease (DKD) is one of the most devastating complications of diabetes mellitus (DM) patients worldwide, with increasing prevalence and high mortality. It accounts for over 50% of individuals receiving dialysis and kidney transplantation therapy 1. Despite the high focus on glomerular changes, tubulointerstitial abnormalities play critical roles in the pathogenesis of DKD, which can be used as indicators to predict early disease progression. It has been established that tubulointerstitial inflammation initiates cell damage and causes renal fibrosis in DKD 2-4. However, the pathogenesis of tubulointerstitial inflammation has not been fully elucidated.

Gut microbiota has been increasingly considered as an important 'organ', comprising 150 times as many genes as the human genome and is implicated in host physiology and pathophysiology 5. Studies conducted to date have provided substantial evidence that dysbiosis is closely correlated with the occurrence and progression of DM and related DKD. Indeed, our previous study revealed that the composition and diversity of fecal microbiota changed significantly in DKD rats and manipulating gut microbiota relieved renal injuries 6. Additionally, increased *Bacteroidotas*: *Firmicutes* ratio was reported in diabetic patients 7-9. Emerging evidence indicates that an imbalanced microbial ecosystem and abnormal bacterial products are involved in tubulointerstitial inflammation. Li *et al.* reported that gut *Akkermansia* abundance and serum level of interleukin (IL)-10 were negatively correlated in chronic kidney disease (CKD) patients 10. Shi *et al.* reported that blood bacterial DNA concentration and plasma levels of C-reactive protein and IL-6 were positively correlated in hemodialysis patients 11. Trimethyl amino oxide, a bacteria-derived uremic toxin, has been shown to exacerbate tubulointerstitial inflammation and renal dysfunction in DKD and obesity-associated CKD 12, 13. In addition, other toxins, such as indoxyl sulfate and p-cresyl sulfate, were reported to trigger inflammatory response in cultured tubular epithelial cells (TECs) and podocytes *in vitro* 14-16. However, at present, the insight into the relevance of host-microbiota interactions in the pathophysiology of DKD is very limited.

Outer membrane vesicles (OMVs) are bacterial extracellular vesicles released by Gram-negative bacteria, ranging from 20 to 250 nm in diameter 17. OMVs contain numerous bacterial components and have been found to mediate host immune response 18. OMVs derived from pulmonary microbes have been shown to induce IL-17 production to promote pulmonary inflammation and fibrosis 19. Recent evidence indicates that gut microbial extracellular vesicles (EVs) can translocate into mammalian circulation and tissues even far beyond the gut and induce tissue inflammation and dysfunction, such as insulin resistance and cognitive impairment 20, 21. Moreover, an *in vitro* study confirmed that OMVs internalized by macrophagocytes delivered lipopolysaccharide (LPS) into the cytoplasm and activated caspase-11/NLRP3-mediated cytokine production 22. Upregulation of renal caspase-11 was reported in DKD mice 23. Also, OMVs derived from gut colonizing bacteria were found in TECs 24. However, whether OMVs contribute to tubulointerstitial inflammation in early DKD remains unknown.

Therefore, this study was designed to investigate the role of OMVs in tubulointerstitial inflammation in the early stage of DKD and its underlying mechanism.

## Materials and Methods

### Diabetic model

Sprague-Dawley rats (male, 8 weeks old, 200-230 g) were housed in specific pathogen free conditions at 22 ℃ with a 12/12-h light/dark cycle and fed *ad libitum* with standard laboratory chow and water. After one-week acclimation, rats were injected intraperitoneally with a single dose of 60 mg/kg streptozotocin (STZ) (Sigma-Aldrich, St. Louis, MO, United States). After 72 h, rats with blood glucose over 16.7 mmol/L were included in the diabetic group (DM). Rats in the control group (Control) were injected with 0.1 mol/L sodium citrate buffer. Control group and DM group rats were given standard drinking water *ad libitum*. All animal experimental protocols used in this study were approved by the Animal Care and Use Committee of Southeast University (Nanjing, China) (NO.20190601006).

### Gut microbiota depletion

Rats were divided into three groups, including Control, DM and DM+AB group. Rats in DM+AB group were given water containing a cocktail of broad-spectrum antibiotics (AB) (0.5 g/L vancomycin, 1 g/L neomycin, 1 g/L metronidazole, 0.1 g/L amphotericin B, 1 g/L ampicillin) *ad libitum* to deplete gut microbiota, while rats in Control and DM groups were given standard drinking water. All the rats were kept for 8 weeks.

### Fecal microbiota transplantation (FMT)

Pooled fresh feces were collected from all healthy donor rats using sterile instruments and homogenized together in sterile phosphate-buffered saline (PBS) with 10% glycerol. The homogenate was passed through stainless steel strainer with 0.25-mm pore size and stored at -80 ℃ until use. After 8-week treatment, the recipient DM rats (DM+FMT) were intravenously injected with omeprazole (50 mg/kg/d) to inhibit gastric acid secretion for 3 days and orally administered with gut microbiota preparations from 6 g feces with a volume of 12 mL once a day for another 3 days using an 80-mm gastric tube. After transplantation, all the rats in Control, DM and DM+FMT groups were maintained for another 8 weeks.

### Fecal bacterial extracellular vesicles (fBEVs) isolation and characterization

At the end of animal raising, 2 g feces from each rat in Control, DM, DM+AB and DM+FMT groups were collected, weighed and homogenized in 35 mL cold PBS. After filtering through a 0.25-mm strainer, the filtrate was centrifuged 3 times at 800×g for 5 min at 4 ℃ to remove insoluble impurity and cell debris. The supernatant was then centrifuged 3 times at 10,000×g for 15 min at 4 ℃ to remove residual bacteria. After multiple centrifugations, the resulting supernatant was collected and filtered through a 0.22-μm cell strainer (Millipore Corporation, Billerica, MA, USA). Then, fBEVs were precipitated by ultracentrifugation at 150,000×g for 2 h at 4 ℃ in a Beckman Optima™ XPN -100 Ultracentrifuge (Beckman Coulter Life Sciences, Indianapolis, IN, USA) using a Type 70Ti rotor. The obtained pellets of fBEVs were each resuspended in 500 μL sterile PBS and the protein contents of the fBEVs samples were assessed using a bicinchoninic acid (BCA) protein assay kit (KeyGen Biotech Co., Ltd., Nanjing, China). The morphology of fBEVs was observed by transmission electron microscopy (TEM) using a HITACHI HT7800 high contrast transmission electron microscope (Hitachi High-tech Co., Tokyo, Japan). Nanoparticle tracking analysis (NTA) was performed to detect the number and size of fBEVs using a Particle Metrix nanoparticle size analyzer ZetaView TWIN (Particle Metrix, Meerbusch, Germany). As for cell intervention *in vitro* and oral administration *in vivo*, fBEVs were extracted from feces of Control and DM rats at week 8.

### Periodic acid Schiff's (PAS) staining

Paraffin-embedded tissue sections were subjected to PAS staining using a PAS stain kit (Wuhan Servicebio Technology Co., Ltd, Wuhan, China) according to the manufacturer's introductions. Briefly, dewaxed sections were immersed in periodic acid for 10 min. After 3 washes, sections were stained with Schiff solution for 30 min and washed. Nuclei were then stained with hematoxylin. The images were acquired using an Olympus optical microscope (Olympus Corporation, Tokyo, Japan) and tubular injury scores were calculated.

### Immunohistochemical and immunofluorescence staining

Tissues embedded in paraffin were sectioned into 5 μm thick slices. Before staining, sections were subjected to dewaxing and heat-induced epitope retrieval. For immunohistochemical staining, sections were blocked with endogenous peroxidase blocking solution, followed by 5% bovine serum albumin. Next, tissues were incubated with appropriate primary antibodies at 4 ℃ overnight and subsequently reacted with corresponding biotin-conjugated secondary antibodies. The color reaction was performed using 3, 3'-diaminobenzidine tetrahydrochloride with hematoxylin counterstaining. Images were acquired with an Olympus optical microscope. For immunofluorescence staining, retrieved tissue sections and fixed cells were permeabilized with 0.25% Triton X-100 in PBS. After blocking, samples were incubated with appropriate primary antibodies and immunoreacted with secondary antibodies conjugated with Alexa Fluor 594 or 488 (1:300; Jackson ImmunoResearch Laboratories Inc., West Grove, PA, USA). 4, 6-diamidino-2-phenylindole (DAPI) dye was used to stain nuclei. Fluorescence images were captured with an Olympus confocal laser scanning microscope (Olympus Corporation). The primary antibodies against the molecules were used: caspase-11 p20 (A-2) (1:50 dilution; sc-374615, Santa Cruz Biotechnology Inc., Santa Cruz, CA, USA), caspase-1/p20/p10 (1:50 dilution; 22915-1-AP, Proteintech Group Inc., Rosemont, IL. USA), anti- IL-1β (1:500 dilution; ab9722, Abcam, Cambridge, USA), anti-tumor necrosis factor-α (TNF-α) (1:100 dilution; ab6671, Abcam), monocyte chemoattractant protein-1 (MCP-1) (1:200 dilution; DF7577, Affinity Biosciences Ltd., Cincinnati, OH, USA), IL-6 (1:300 dilution; DF6087, Affinity Biosciences Ltd.), zona occludens 1 (ZO-1) (C-19) (1:50 dilution; sc-8146, Santa Cruz Biotechnology Inc.), occludin (H-279) (1:50 dilution; sc-5562, Santa Cruz Biotechnology Inc.), Anti-lipid A (1:10 dilution; ab8467, Abcam), Anti-outer membrane protein F (ompF) (1:200 dilution; bs-2086R, Bioss Antibodies Inc., Woburn, MA, USA), Anti-aquaporin 1 (AQP-1) (1:100 dilution; ab9566, Abcam). Also, fluorescein-labeled lotus tetragonolobus lectin (LTL; 1:200 dilution; FL-1321-2) was obtained from Vector Laboratories Inc. (Burlingame, CA, USA).

### Cell culture

Human kidney-2 (HK-2) cells were cultured in Dulbecco's Modified Eagle's medium/nutrient mixture F-12 containing 10% fetal bovine serum and 1% penicillin /streptomycin at 37 ℃ with 5% CO_2_. For stimulation experiments, HK-2 cells were treated with 5 μg/mL fBEVs. To elucidate the mechanism of fBEVs-induced tubulointerstitial inflammation, specific small interfering RNA (siRNA) against *CASP4* was used to knockdown the expression of caspase-4. And 30 μg/mL polymyxin B (PMB) was used to neutralize LPS in fBEVs. Each experiment was repeated at least three times and conducted in triplicates.

### Assay of fBEVs internalization

DM-fBEVs were labeled with Dil dye (a fluorescent lipophilic cationic indocarbocyanine dye) at 37 ℃ for 30 min, termed as Dil-fBEVs. To investigate the internalization mechanism, HK-2 cells were pre-incubated with or without various endocytosis inhibitors for 1 h, including 1 μg/mL Cytochalasin D (Cyto), 200 μmol/L Dansylcadaverine (Dan), 80 μmol/L Dynasore (Dyna) and 50 μmol/L Nystatin (Nys). Then, the medium was changed and supplemented with 5 μg/mL Dil-fBEVs. After co-culture for 24 h, cells were observed. Images were acquired with an Olympus confocal laser scanning microscope. The quantification of fluorescent dots in cells was analyzed using the Image J software (National Institute of Health, Bethesda, MD, USA).

### Cytosol extraction and limulus amebocyte lysate (LAL) assay

HK-2 cells were plated in 6-well culture plates and exposed to medium with or without 5 μg/mL fBEVs from Control or DM group for 24 h. After washing 4 times with cold sterile PBS, the cytosol fraction of HK-2 cells was extracted using a digitonin-based method as previously described 25. The digitonin reagent consisted of 0.010% digitonin, 10 mmol/L HEPES pH 7.4, 300 mmol/L sucrose, 100 mmol/L NaCl, 3 mmol/L MgCl_2_, 5 mmol/L EDTA and 1× complete protease inhibitors. A 300 μL volume of digitonin reagent was added to each well and the plate was placed on ice for 8 min. Then, the supernatant was collected by tilting the plates and centrifuged at 1,000×g for 5 min at 4 ℃ to remove dead cells. The cytosol-containing supernatants were subjected to the LAL assay using the Pierce^TM^ Chromogenic Endotoxin Quant Kit (Pierce. Biotechnology, Rockford, IL, USA) to quantify LPS according to the manufacturer's introductions. In addition, the BCA protein assay was performed to measure protein concentration.

### DM-fBEVs-treated mice model

Eight-week-old C57BL/6J mice were randomly assigned to two groups: the DM-fBEVs group orally gavaged with 200 μg DM-fBEVs in 200 μL PBS once every two days for 16 weeks and the Control group orally gavaged with an equal volume of PBS. Prior to gavage, mice were starved for 5 h to reduce the production of gastric acid. Ultimately, urine and serum samples were collected. Organs were harvested for further analysis.

### *In vivo* biodistribution assay of DM-fBEVs

DM-fBEVs were stained with DiD dye (a far-red fluorescent lipophilic cationic indocarbocyanine dye). According to previous studies with some modifications 21, 24, mice were orally treated with 200 μL PBS or DiD-fBEVs (200 μg) for 5 days and intraperitoneally injected with 2.5 mg/kg LPS in the last two days to increase the gut permeability. Eventually, organs were removed and observed using an IVIS^®^ Spectrum Imaging System (PerkinElmer Inc., Waltham, MA, USA). Additionally, kidney tissues embedded in optimal cutting temperature medium were sectioned and observed with a confocal laser scanning microscope.

### Assay of serum cytokines

Rat serum levels of cytokines were measured using the appropriate enzyme linked immunosorbent assay (ELISA) kits (Elabscience Biotechnology Co., Ltd, Wuhan, China) in accordance with the manufacturer's instructions. Briefly, blood was collected and serum was obtained by centrifugation at 3,000×rpm for 15 min at 4 ℃. Serum samples together with standard solutions were pipetted into microplates. Then, biotinylated detection antibodies, horse radish peroxidase (HRP) conjugate, wash buffer, substrate reagent and stop solution were added step by step. The plates were incubated at 37 ℃ and the OD values were read at the wavelength of 450 nm.

Mouse serum cytokines were determined with a mouse premixed multi-analyte kit using a Luminex liquid system (Luminex Corporation, Austin, TX, USA). First, beads coated with specific antibodies were added to the wells of 96-well plates. Samples, blank and standards were added to the corresponding wells of the plates and incubated, followed by the addition of the biotin antibody cocktail, wash buffer and streptavidin-phycoerythrin conjugate. Results were obtained by measuring the luminescence on a Luminex 200 system (Luminex Corporation).

### Urine biochemistry test

After collection, urine was centrifugated at 3,000×rpm for 15 min to obtain debris free samples. N-acetyl-β-D-glucosaminidase (NAG) was determined using a colorimetric method. Creatinine was measured with the ammonia iminohydrolase method.

### Quantitative real-time polymerase chain reaction (qRT-PCR) analysis

Total RNA was extracted from cells using Trizol reagent (Takara Bio Inc., Kusatsu, Shiga, Japan) and reverse-transcribed into cDNA with the PrimeScript RT Master Mix (Takara Bio Inc.). PCR amplification was performed in a 10 µL reaction volume using SYBR Premix Ex Tag^TM^ (Takara Bio Inc.). *GAPDH* was used as an internal reference. The relative gene expression level was quantified using the 2^-ΔΔCT^ method 26. Primer sequences are listed in Table S1.

### Western blot analysis

Tissues or cells were lysed in radioimmunoprecipitation assay buffer containing protease inhibitors. Lysates were boiled with loading buffer and dithiothreitol at 75 ℃ for 10 min. The protein samples were then separated by sodium dodecyl sulfate polyacrylamide gel electrophoresis and transferred to a polyvinylidene fluoride membrane. After blocking the membrane with the appropriate buffer, the membrane was probed by incubation overnight at 4 ℃ with the corresponding primary antibodies: caspase-11 p20 (A-2) (1:500 dilution; sc-374615, Santa Cruz Biotechnology Inc.), caspase-4/p20/p10 (1:500 dilution; 11856-1-AP; Proteintech Group Inc.), caspase-1 p20 (1:500 dilution; sc-398715, Santa Cruz Biotechnology Inc.), caspase-1/p20/p10 (1:500 dilution; 22915-1-AP, Proteintech Group Inc.), Anti-IL-1β (1:1000 dilution; ab9722, Abcam), Anti-TNF-α (1:1000 dilution; ab6671, Abcam), MCP-1 (1:1000 dilution; DF7577, Affinity Biosciences Ltd.), IL-6 (1:1000 dilution; DF6087, Affinity Biosciences Ltd.), Anti-lipid A (1:200 dilution; ab8467, Abcam). Membranes were washed 3 times with 0.1% Tris-buffered saline Tween and incubated with the appropriate HRP-conjugated secondary antibodies. Signals were detected by electrochemiluminescence assay using the SuperSignal™ West Atto Ultimate Sensitivity Chemiluminescent Substrate (Thermo Fisher Scientific Inc., Waltham, MA, USA). For fBEVs immunoblotting, fBEVs were extracted from feces in the same weight from different groups and resuspended in equal volume of PBS. Then, fBEVs in equal suspensions were lysed isometrically and were probed with Anti-ompF (1:1000 dilution; bs-2086R, Bioss Antibodies).

### Statistical analysis

Data are presented as the mean ± SD. SPSS software was used to perform analysis. For data with normality, multigroup statistical significance was determined by one-way analysis of variance, while an unpaired t test was used between two groups. Statistical parameters are all indicated in the figure legends.

## Results

### Gut microbiota is involved in systemic and tubulointerstitial inflammation in diabetic rats

**Figure 1:**
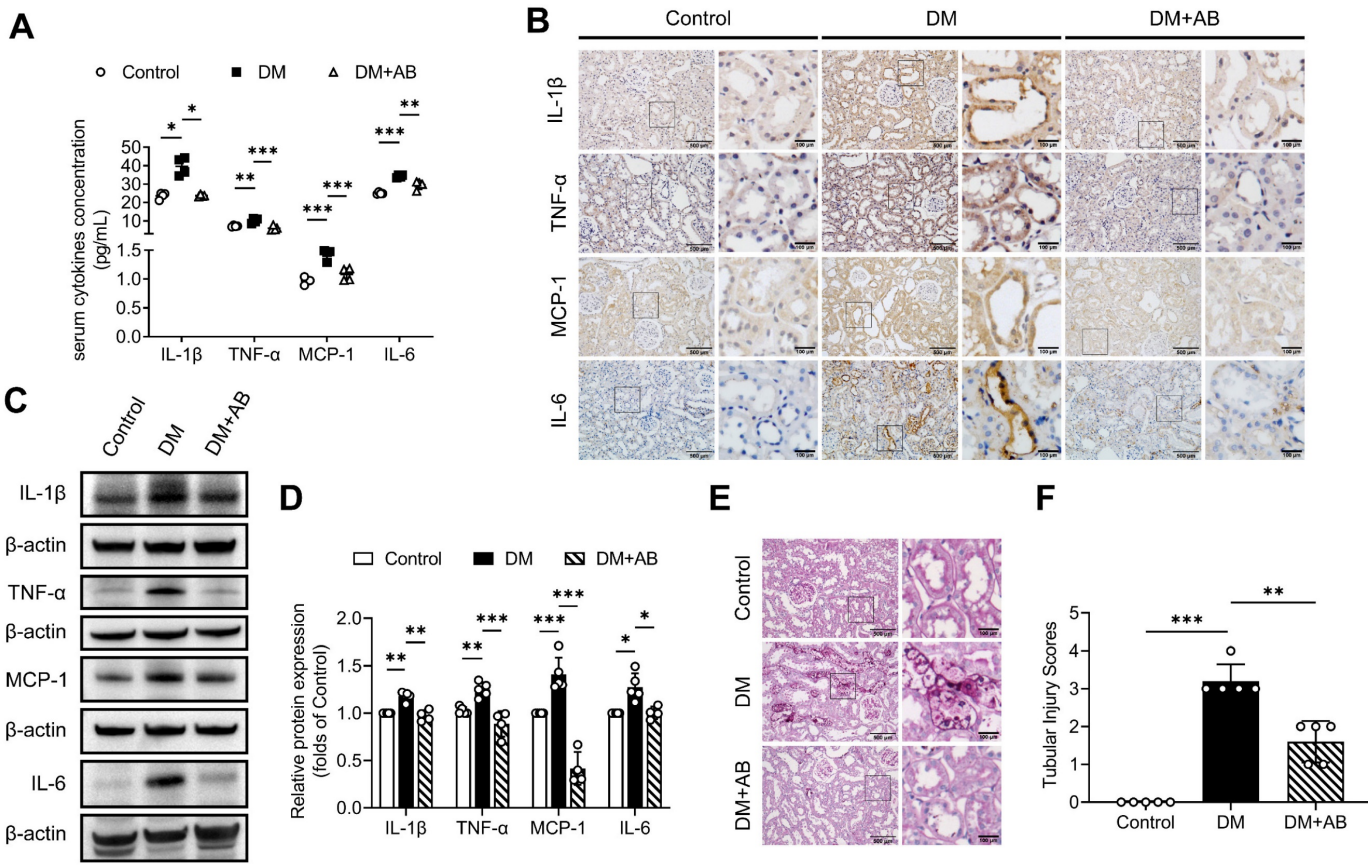
** Gut microbiota depletion attenuates systemic and tubulointerstitial inflammation in diabetic rats.** Gut microbiota of DM rats was depleted by treatment with broad-spectrum antibiotics administered in the drinking water (DM+AB). **(A)** Serum inflammatory cytokines, including IL-1β, TNF-α, MCP-1 and IL-6, were measured by ELISA (n=4). **(B)** Tubulointerstitial inflammation was detected by immunohistochemical staining (scale bar, 500 μm and 100 μm, original magnification × 200). **(C-D)** Expression levels of inflammatory proteins in renal cortex were detected by Western blot analysis and quantified (n=4-5). **(E-F)** Histopathological injuries of tubulointerstitium were evaluated by PAS staining (scale bar, 500 μm and 100 μm, original magnification × 200) and quantified (n=5). *P < 0.05; **P < 0.01; ***P < 0.001.

**Figure 2:**
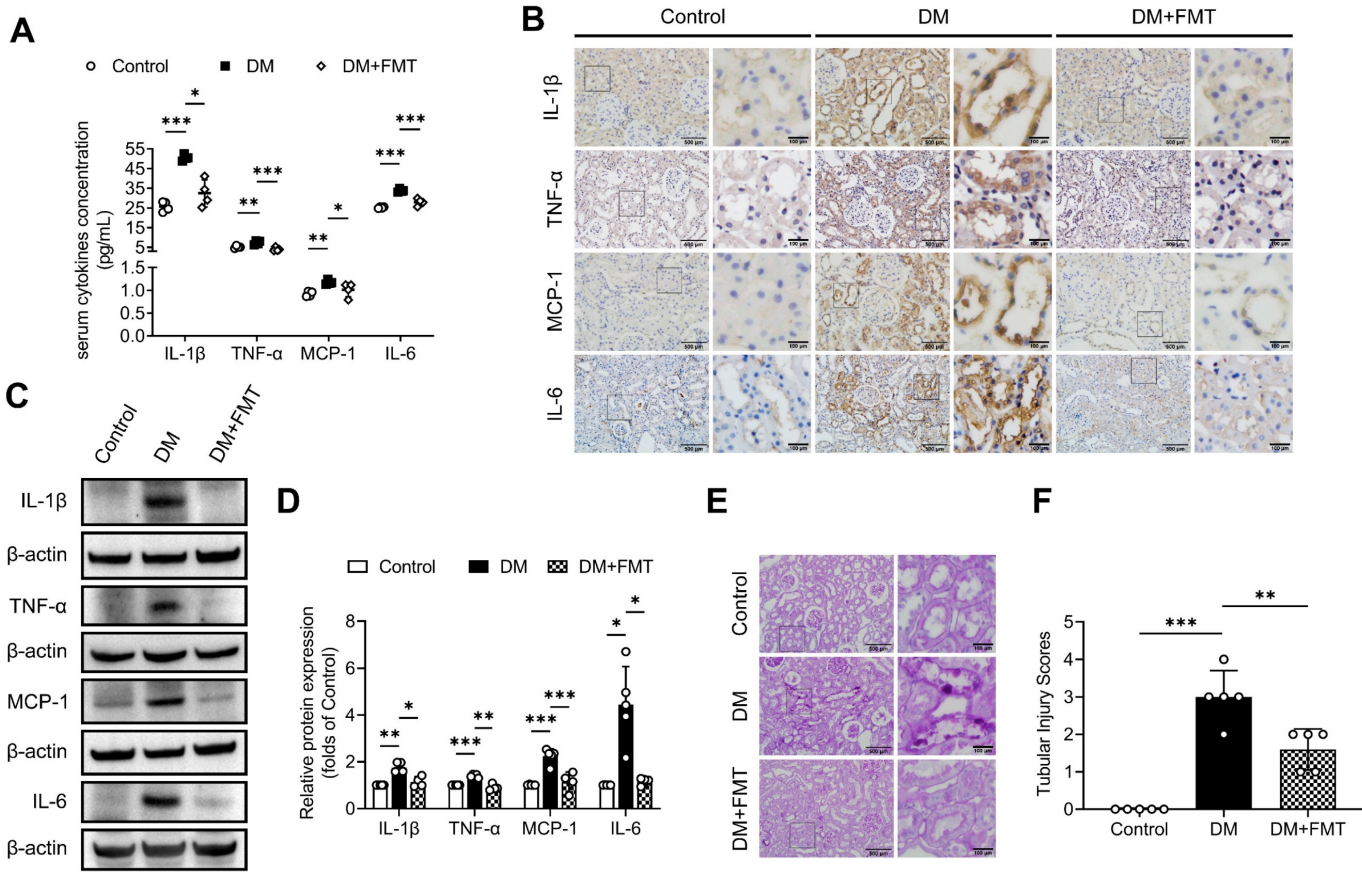
** Normal fecal microbiota transplantation attenuates systemic and local tubulointerstitial inflammation in diabetic rats.** Fecal microbiota extracted from normal control group was orally transplanted into recipient rats in the DM group (DM+FMT). **(A)** Serum inflammatory cytokines including IL-1β, TNF-α, MCP-1 and IL-6, were measured by ELISA (n=4). **(B)** Tubulointerstitial inflammation was detected by immunohistochemical staining (scale bar, 500 μm and 100 μm, original magnification × 200). **(C-D)** Expression levels of inflammatory proteins in renal cortex were detected by Western blot analysis and quantified (n=4-5). **(E-F)** Histopathological injuries of tubulointerstitium were evaluated by PAS staining (scale bar, 500 μm and 100 μm, original magnification × 200) and quantified (n=5). *P < 0.05; **P < 0.01; ***P < 0.001.

To investigate whether gut microbiota is involved in tubulointerstitial inflammation in the context of DKD, we first treated STZ-induced diabetic rats with broad-spectrum antibiotics (AB) in drinking water to deplete their microbiota. As demonstrated by our previous study 6, gut microbiota composition with declining diversity and relative abundance in DM group differed from that in normal group and AB treatment eliminated most microbiota. As shown in Figure 1A, depletion of the microbiota in diabetic rats downregulated serum levels of inflammatory cytokines, such as IL-1β, TNF-α, MCP-1 and IL-6. We further evaluated the effects of microbiota on tubulointerstitial changes. We found that enhanced tubulointerstitial inflammation and upregulated inflammatory protein expressions in renal cortex tissues in diabetic rats were alleviated after treatment with the AB (Figure 1B-D). PAS staining also revealed that tubular injuries in diabetic rats, characterized by glycogen deposition, swelling, abscission and necrosis of tubule cells, were alleviated after administration of the AB (Figure 1E-F). To further examine the role of microbiota in inflammation, diabetic rats were transplanted with fecal microbiota from healthy donor rats of the control group. Consistent with the AB treatment, normal fecal microbiota transplantation (FMT) improved serum levels of inflammatory cytokines and tubular inflammation (Figure 2A-D) and alleviated tubular pathological injuries in diabetic rats (Figure 2E-F). These findings indicate that gut microbiota plays critical roles in systemic and tubulointerstitial inflammation in DKD and modulating gut microbiota can rescue these changes.

### Gut microbiota-derived OMVs are increased in diabetic rats

**Figure 3:**
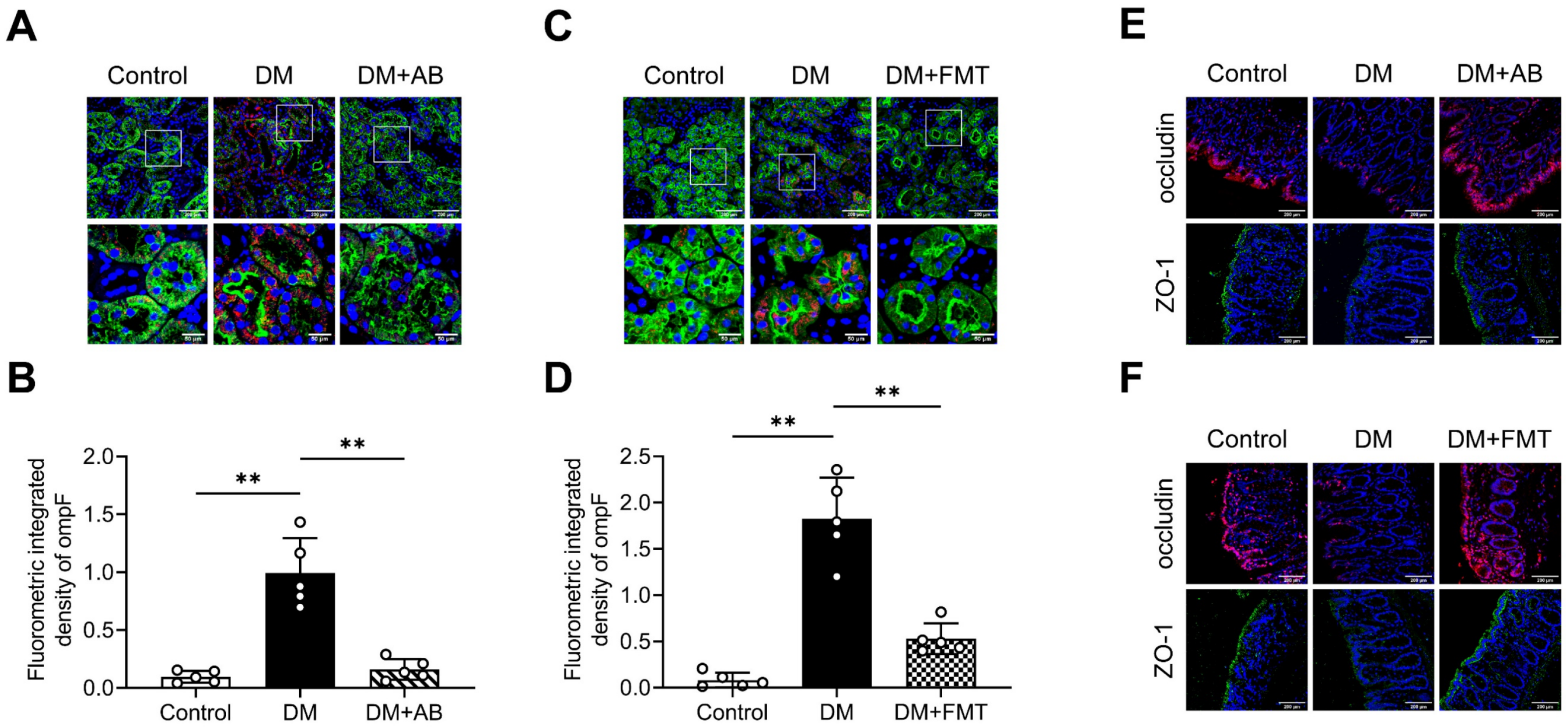
** Gut microbiota-derived OMVs cross the impaired gut-vascular barrier and accumulate in the tubulointerstitium of DM rats. (A-D)** The presence of ompF^+^ OMVs (red dots) in renal tubular marked with AQP-1 (green) was detected by immunofluorescence staining and quantified (n=5). Nuclei were stained with DAPI (blue) (scale bar, 200 μm and 50 μm, original magnification × 400). **(E-F)** Expressions of tight junction proteins occludin (red) and ZO-1 (green) in colon tissues were detected by immunofluorescence staining. Nuclei were stained with DAPI (blue) (scale bar, 200 μm, original magnification × 400). **P < 0.01.

OMVs mediate microbiota-host interactions and are involved in immune modulation 18. Thus, we investigated the possible OMVs changes in diabetic rats. We detected OMVs infiltration in tubulointerstitium of rats by staining ompF, an OMVs-associated porin protein. The results showed that larger quantities of ompF^+^ OMVs were found infiltrated in the tubulointerstitium of diabetic rats compared with control rats, which were decreased by AB and FMT treatment (Figure 3A-D). As previously reported 27, gut integrity was impaired in the DM group, as indicated by the reduction of the tight junction-associated proteins occludin and ZO-1, which were improved after AB and FMT treatment (Figure 3E-F). These findings suggest that gut microbiota-derived OMVs are increased in diabetic rats and may be transported through disrupted gut-vascular barrier into the tubulointerstitium of diabetic rats.

**Figure 4:**
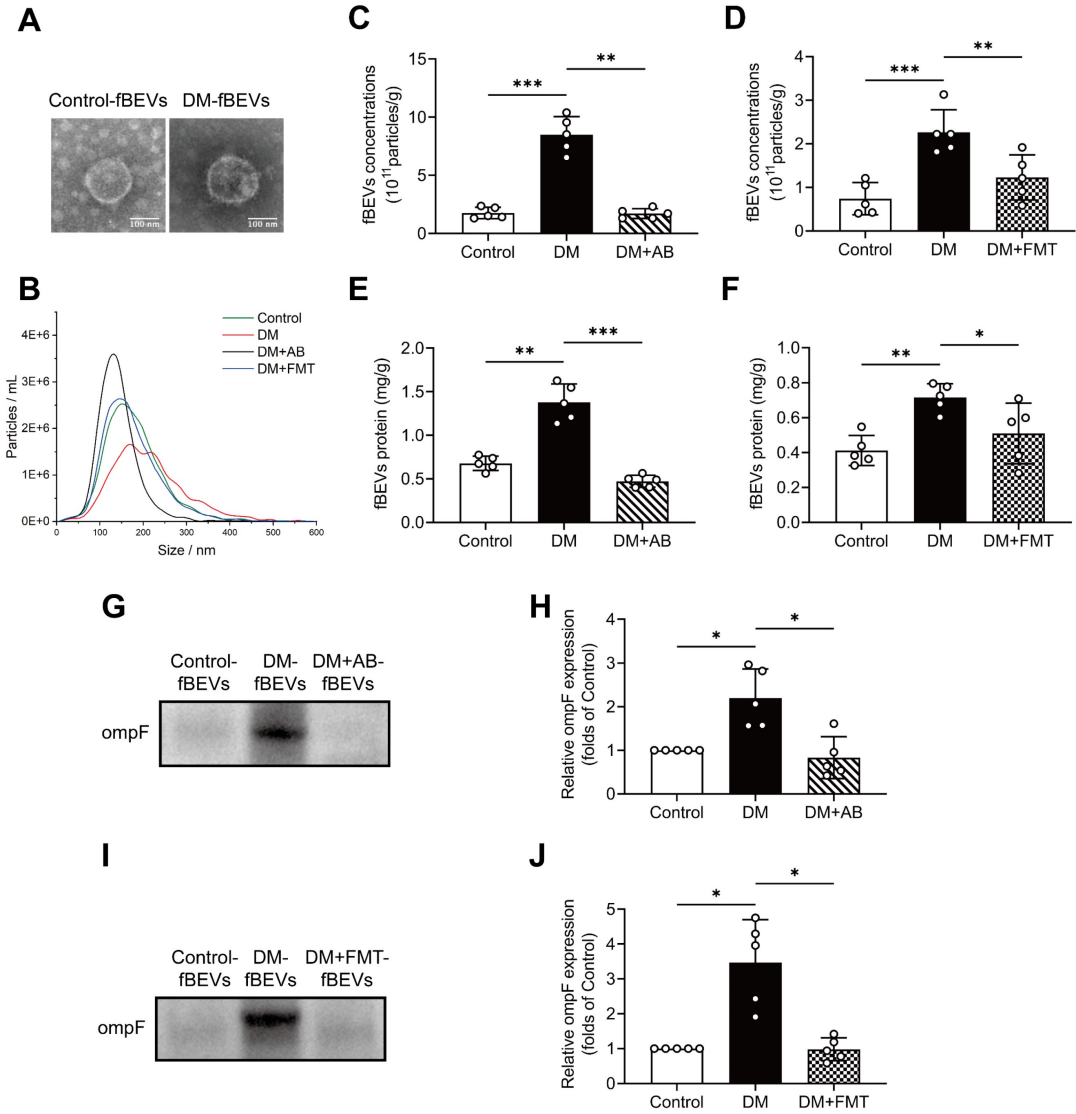
** The extraction and analysis of fBEVs.** Stools in the same weight from Control, DM, DM+AB and DM+FMT rats were collected to extract fBEVs, which were resuspended in equal volumes of PBS. **(A)** TEM was applied to observe fBEVs (scale bar, 100 nm, original magnification × 40k). **(B)** NTA was used to determine the size distribution of fBEVs from different groups. (C-D) Concentrations of fBEVs were quantified by the NTA (n=5). **(E-F)** Total protein of fBEVs was quantified by the BCA protein assay (n=5). **(G-J)** The outer membrane protein ompF in equal volumes of purified fBEVs suspension was measured by Western blot analysis to determine the amount of OMVs in the gut (n=5). *P < 0.05; **P < 0.01; ***P < 0.001.

To further validate the changing amount of OMVs, fBEVs were isolated and characterized by TEM observation and NTA. As shown, control group-derived fBEVs (Control-fBEVs) and DM group-derived fBEVs (DM-fBEVs) displayed spherical structure with diameters of 181.1±74.3 nm and 215.2±91.4 nm, respectively (Figure 4A-B). Additionally, NTA revealed that the number of DM-fBEVs was significantly increased relative to Control-fBEVs in the same weight of feces (Figure 4C-D). AB as well as FMT treatment reduced fBEVs concentrations (Figure 4C-D). Also, since the protein level is often used to quantify the number of EVs, the BCA protein assay was performed and confirmed that the total protein of DM-fBEVs suspension was much higher than that of Control-fBEVs, which was reduced by AB and FMT treatment (Figure 4E-F). In addition, it was also found that the level of ompF protein in fBEVs suspension was markedly upregulated in the DM group, suggesting that there were much more OMVs in the feces of diabetic rats (Figure 4G-J). Consistently, both AB and FMT treatment downregulated ompF protein levels (Figure 4G-J). These results further indicate that the amount of OMVs produced in the DM group is increased, which may be involved in the pathogenesis and progression of DKD.

### Diabetic gut microbiota-derived OMVs induce tubulointerstitial inflammation and kidney injury

**Figure 5:**
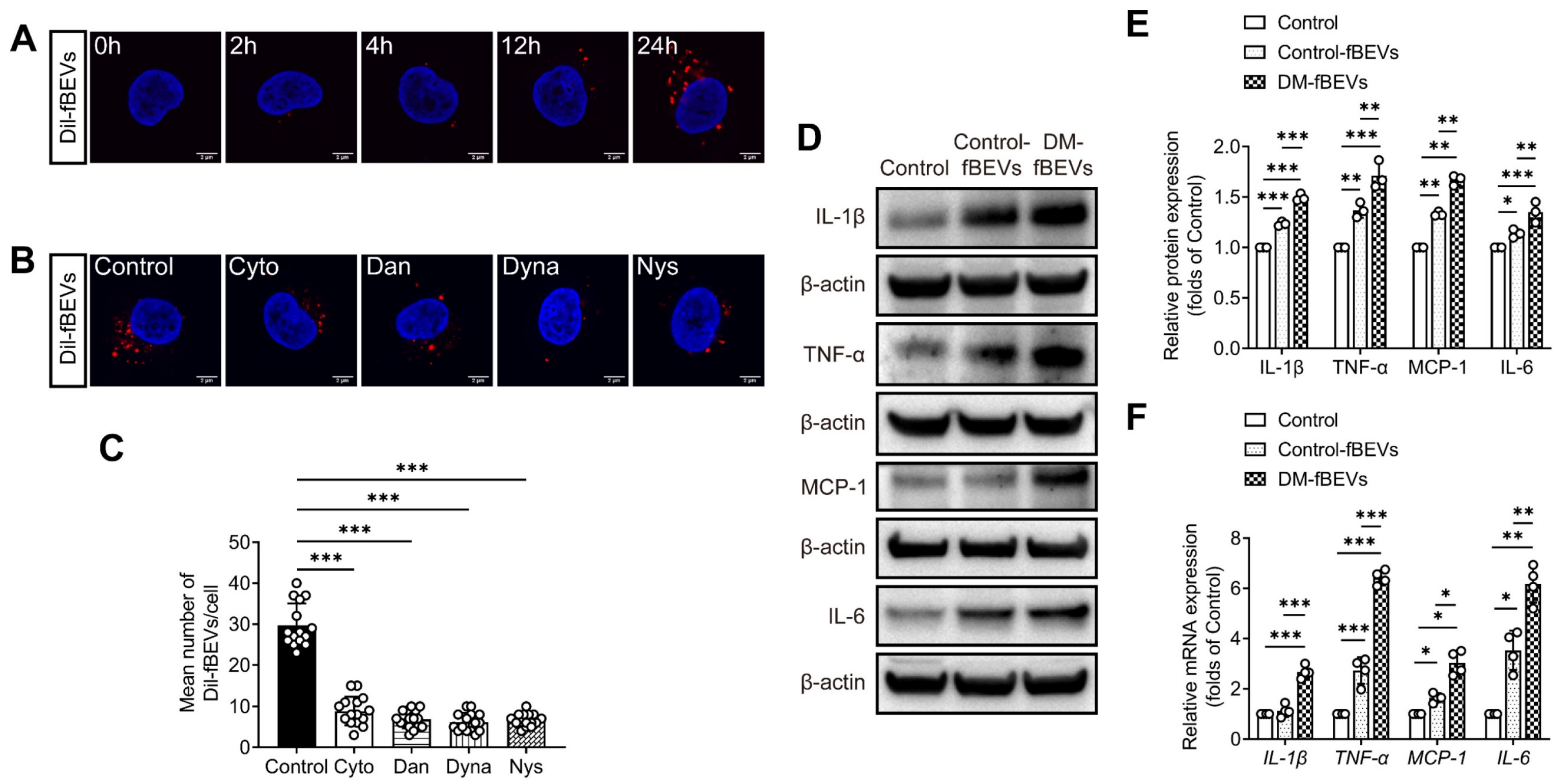
** DM-fBEVs are endocytosed by HK-2 cells and directly promote inflammation *in vitro***. **(A)** HK-2 cells were incubated with Dil-conjugated DM-fBEVs for 0, 2, 4, 12, 24 h. The uptake of fBEVs was observed by confocal laser scanning microscopy (scale bar, 2 μm, original magnification × 1000). **(B-C)** HK-2 cells were preincubated without or with inhibitors for 1 h and exposed to 5 μg/mL Dil-conjugated DM-fBEVs for 24 h. Cellular fBEVs were quantified (n=3, 5 pictures/group/experiment) (scale bar, 2 μm, original magnification × 1000). Control, cells without inhibitor treatment; Cyto, Cytochalasin D (1 μg/mL); Dan, Dansylcadaverine (200 μmol/L); Dyna, Dynasore (80 μmol/L); Nys, Nystatin (50 μmol/L). HK-2 cells were incubated with 5 μg/mL Control-fBEVs or DM-fBEVs for 24 h. **(D-E)** Protein (n=3) levels of IL-1β, TNF-α, MCP-1 and IL-6 were detected by Western blot analysis. **(F)** mRNA (n=4) levels of *IL-1β*, *TNF-α*, *MCP-1* and *IL-6* were measured by qRT-PCR analysis. *P < 0.05; **P < 0.01; ***P < 0.001.

To determine whether microbiota could communicate with TECs through OMVs and induce tubulointerstitial inflammation in DKD, we first examined whether OMVs could be internalized by TECs *in vitro*. DM-fBEVs were labeled with the Dil dye and added to cultures of HK-2 cells, incubating for 0, 2, 4, 12 and 24 h. As shown by the red fluorescence signals in the cytoplasm of cells, DM-fBEVs were markedly taken up by HK-2 cells after 24-h incubation (Figure 5A). Previous studies have reported that bacterial OMVs can enter host cells by various endocytosis pathways. To determine whether the uptake of DM-fBEVs into HK-2 cells was mediated by endocytic pathways, HK-2 cells were incubated with Dil-fBEVs in the absence (Control) or presence of various endocytosis inhibitors, including Cytochalasin D (Cyto, a micropinocytosis and membrane fusion inhibitor), Dansylcadaverine (Dan, a receptor-mediated endocytosis inhibitor), Dynasore (Dyna, a clathrin and caveolin mediated endocytosis inhibitor) and Nystatin (Nys, a caveolin-mediated endocytosis and lipid raft inhibitor). We found that all inhibitors dramatically blocked the uptake of fBEVs (Figure 5B-C), strongly suggesting that the uptake of fBEVs by TECs is mediated by endocytosis pathways. Also, our analysis of inflammatory markers in HK-2 cells revealed that DM-fBEVs more significantly enhanced the cellular expression of IL-1β, TNF-α, MCP-1 and IL-6 than Control-fBEVs (Figure 5D-F).

**Figure 6:**
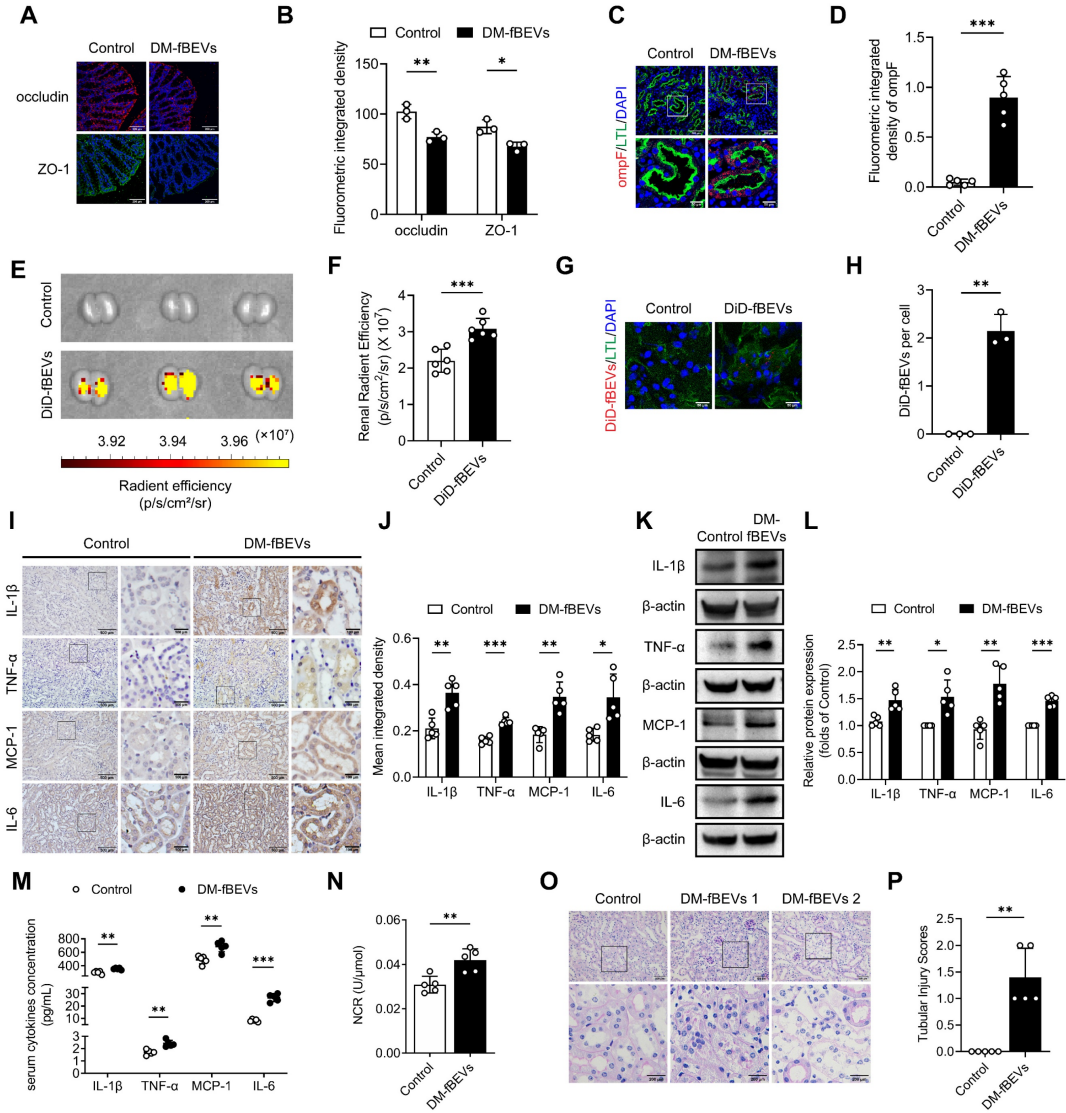
** Orally administered DM-fBEVs translocate into tubulointerstitium and induce tubulointerstitial inflammation and kidney injuries**. **(A-B)** Expressions of tight junction proteins occludin (red) and ZO-1 (green) in mouse colon tissues were determined by immunofluorescence staining and quantified (n=3). Nuclei were stained with DAPI (blue) (scale bar, 200 μm, original magnification × 400). **(C-D)** The presence of ompF^+^ OMVs (red dots) in mouse tubulointerstitium marked with LTL (green) was determined by immunofluorescence staining and quantified (n=5). Nuclei were stained with DAPI (blue) (scale bar, 200 μm and 50 μm, original magnification × 400). **(E-F)** DiD-fBEVs biodistribution in kidney was assessed using an IVIS Spectrum Imaging System and quantified (n=6). **(G-H)** Renal DiD-fBEVs signals (red) were detected by confocal laser scanning microscopy and quantified (n=3). Proximal tubules were stained with LTL (green). Nuclei were stained with DAPI (blue) (scale bar, 50 μm, original magnification ×400). **(I-J)** Tubulointerstitial inflammation in mice was detected by immunohistochemical staining and quantified (n=5) (scale bar, 500 μm and 100 μm, original magnification × 200). **(K-L)** Expression levels of inflammatory proteins in mouse renal cortex were detected by Western blot analysis (n=5). **(M)** Concentrations of serum cytokines, including IL-1β, TNF-α, MCP-1 and IL-6, were measured by the Luminex assay (n=5). **(N)** Urinary NCR was measured in mice (n=5). **(O-P)** Tubular pathological injuries were detected by PAS staining (scale bar, 500 μm and 200 μm, original magnification × 400) and quantified (n=5). DM-fBEVs 1 and 2 indicate images from two mice. *P < 0.05; **P < 0.01; ***P < 0.001.

We also studied the effects of bacterial particles *in vivo* in mice orally administered with DM-fBEVs once every two days for 16 weeks. Tissue immunofluorescence staining revealed disrupted gut integrity (Figure 6A-B) and more OMVs translocated into tubulointerstitium of mice administered with DM-fBEVs (Figure 6C-D). In addition, the *in vivo* biodistribution assay detected DiD-fBEVs signal in kidney after oral administration (Figure 6E-F), which was also confirmed by confocal laser scanning microscopy imaging, showing red immunofluorescence spots in the cytoplasm of tubular cells (Figure 6G-H). Oral gavage administration of DM-fBEVs efficiently induced inflammatory responses in tubulointerstitium (Figure 6I-L), coupled with an increase in the serum levels of inflammatory cytokines (Figure 6M). Also, the urine NAG-creatinine ratio (NCR) was elevated after DM-fBEVs treatment (Figure 6N). Aggravated tubulointerstitial injuries were also observed (Figure 6O-P). This study also provide evidence that DM-fBEVs, continuously administrated orally, can damage the gut mucosal barrier and promote their dissemination far away into the tubulointerstitium, thereby triggering tubulointerstitial inflammation and leading to morphological and functional abnormalities in kidney tissue.

### The caspase-11 pathway is involved in OMV-induced tubulointerstitial inflammation

**Figure 7:**
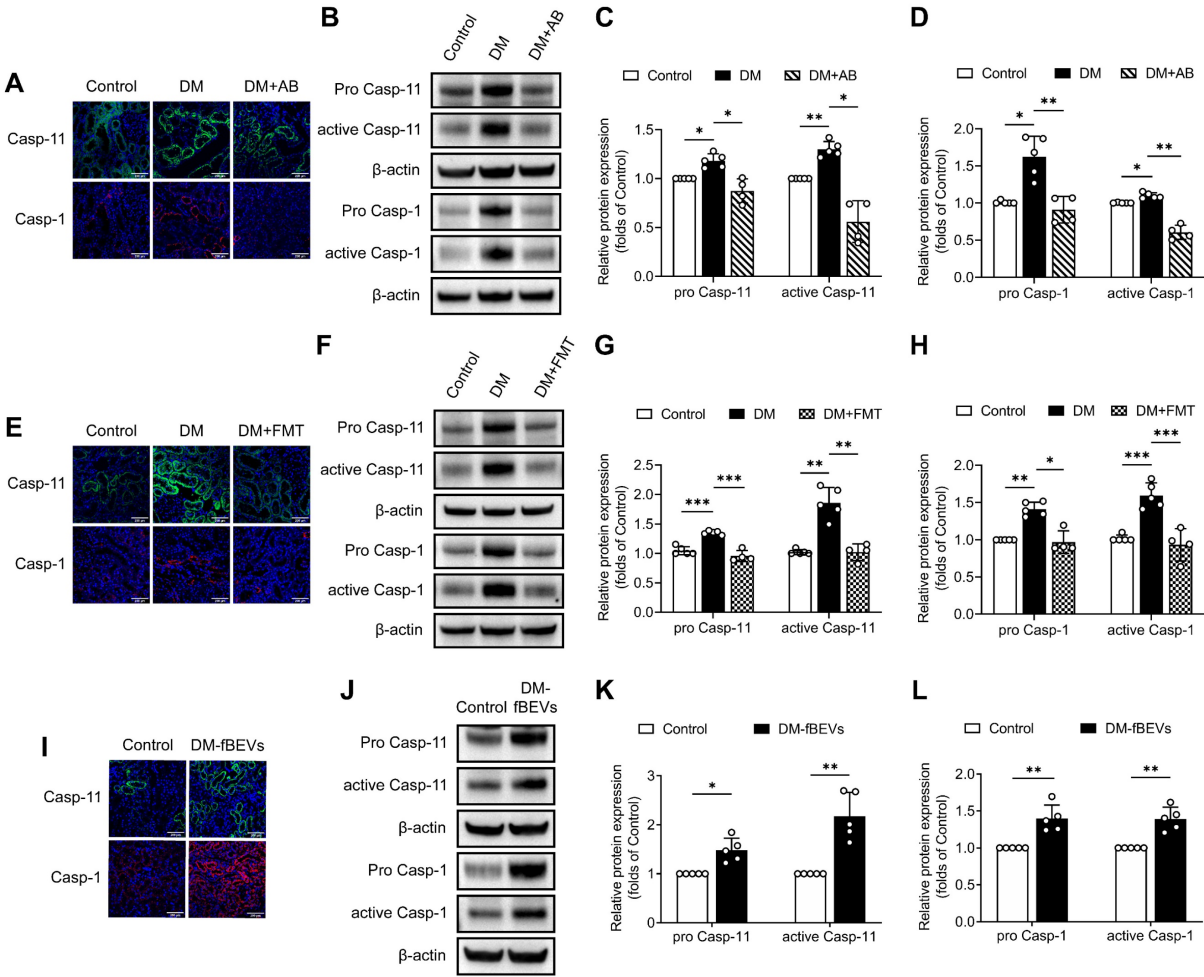
** DM-fBEVs mediate tubulointerstitial inflammation by activating the caspase-11/caspase-1 pathway *in vivo***. **(A)** Expression levels of caspase-11 and caspase-1 in tubulointerstitium of rats were determined by immunofluorescence staining. Nuclei were stained with DAPI (blue) (scale bar, 200 μm, original magnification × 400). **(B-D)** Protein expression levels of pro caspase-11, active caspase-11, pro caspase-1 and active caspase-1 in the renal cortex of rats were detected by Western blot analysis (n=4-5). **(E)** Expression levels of caspase-11 and caspase-1 in tubulointerstitium of rats were determined by immunofluorescence staining. Nuclei were stained with DAPI (blue) (scale bar, 200 μm, original magnification × 400). **(F-H)** Protein expression levels of pro caspase-11, active caspase-11, pro caspase-1 and active caspase-1 in the renal cortex of rats were detected by Western blot analysis (n=4-5). **(I)** Expression levels of caspase-11 and caspase-1 in tubulointerstitium of mice were determined by immunofluorescence staining. Nuclei were stained with DAPI (blue) (scale bar, 200 μm, original magnification × 400). **(J-L)** Protein expression levels of pro caspase-11, active caspase-11, pro caspase-1 and active caspase-1 in the renal cortex of mice were detected by Western blot analysis (n=5). *P < 0.05; **P < 0.01; ***P < 0.001.

**Figure 8:**
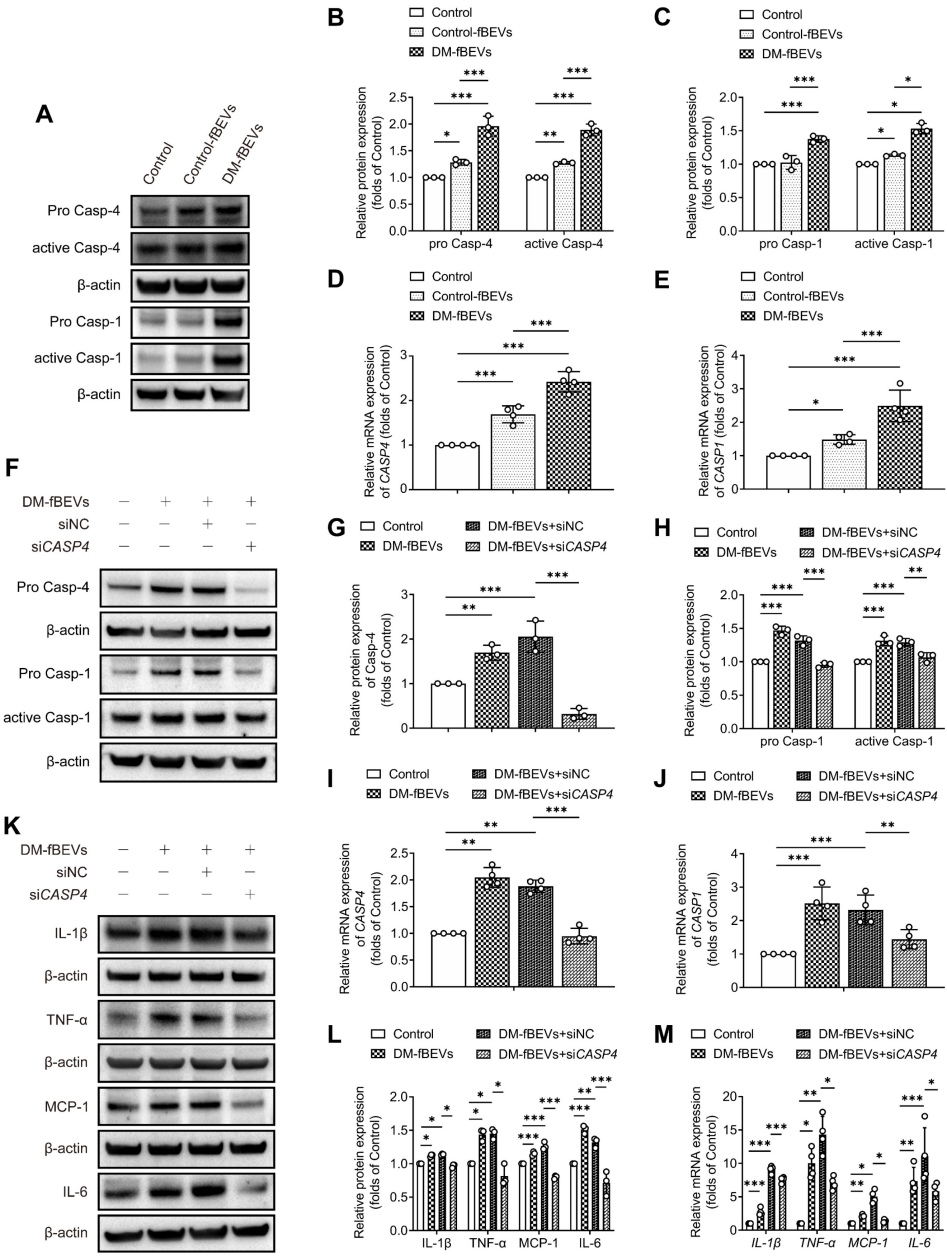
** DM-fBEVs mediate tubulointerstitial inflammation by activating the caspase-11/caspase-1 pathway *in vitro***. *In vitro*, HK-2 cells were treated without (Control) or with 5 μg/mL Control-fBEVs or DM-fBEVs for 24 h. **(A-C)** Protein expression levels of pro caspase-4, active caspase-4, pro caspase-1 and active caspase-1 in HK-2 cells were detected by Western blot analysis (n=3). **(D-E)** mRNA expression levels of *CASP4* and *CASP1* in HK-2 cells were measured by qRT-PCR analysis (n=4). The expression of caspase-4 in HK-2 cells was knocked down with siRNA against *CASP4* (siNC, negative control siRNA; si*CASP4*, *CASP4* siRNA). **(F-H)** Protein expression levels of pro caspase-4, pro caspase-1 and active caspase-1 were determined by Western blot analysis (n=3). **(I-J)** mRNA expression levels of *CASP4* and *CASP1* were measured by qRT-PCR analysis (n=4). **(K-L)** Protein expression levels of IL-1β, TNF-α, MCP-1 and IL-6 were detected by Western blot analysis (n=3). **(M)** mRNA expression levels of *IL-1β*, *TNF-α*, *MCP-1* and *IL-6* were measured by qRT-PCR analysis (n=4). *P < 0.05; **P < 0.01; ***P < 0.001.

We further investigated the mechanism through which OMVs communicate with the host kidney. Immunofluorescence staining and Western blot analysis of rat renal tissues revealed that the expression levels of pro caspase-11, active caspase-11, pro caspase-1 and active caspase-1 were significantly increased in diabetic rats, an effect that was reversed by AB and FMT treatment (Figure 7A-H). Consistent with these findings, oral gavage administration of DM-fBEVs also upregulated the expression levels of pro caspase-11, active caspase-11, pro caspase-1 and active caspase-1 in mouse tubulointerstitium (Figure 7I-L). Caspase-4 in humans is a homologue of caspase-11. In the *in vitro* study, DM-fBEVs more markedly upregulated the expression levels of pro caspase-4, active caspase-4, pro caspase-1 and active caspase-1 in HK-2 cells, compared with Control-fBEVs (Figure 8A-E). In order to confirm the role of the caspase-11 pathway in OMVs-mediated inflammation in TECs, siRNA against *CASP4* was used to knockdown caspase-4 expression in HK-2 cells. The results revealed that caspase-4 knockdown inhibited the activation of caspase-1 (Figure 8F-J) and the inflammatory response (Figure 8K-M) induced by DM-fBEVs, increasing the evidence for the involvement of the caspase-11 pathway in OMVs-initiated tubulointerstitial inflammation in DKD.

### OMVs-derived LPS promotes inflammation in TECs

**Figure 9:**
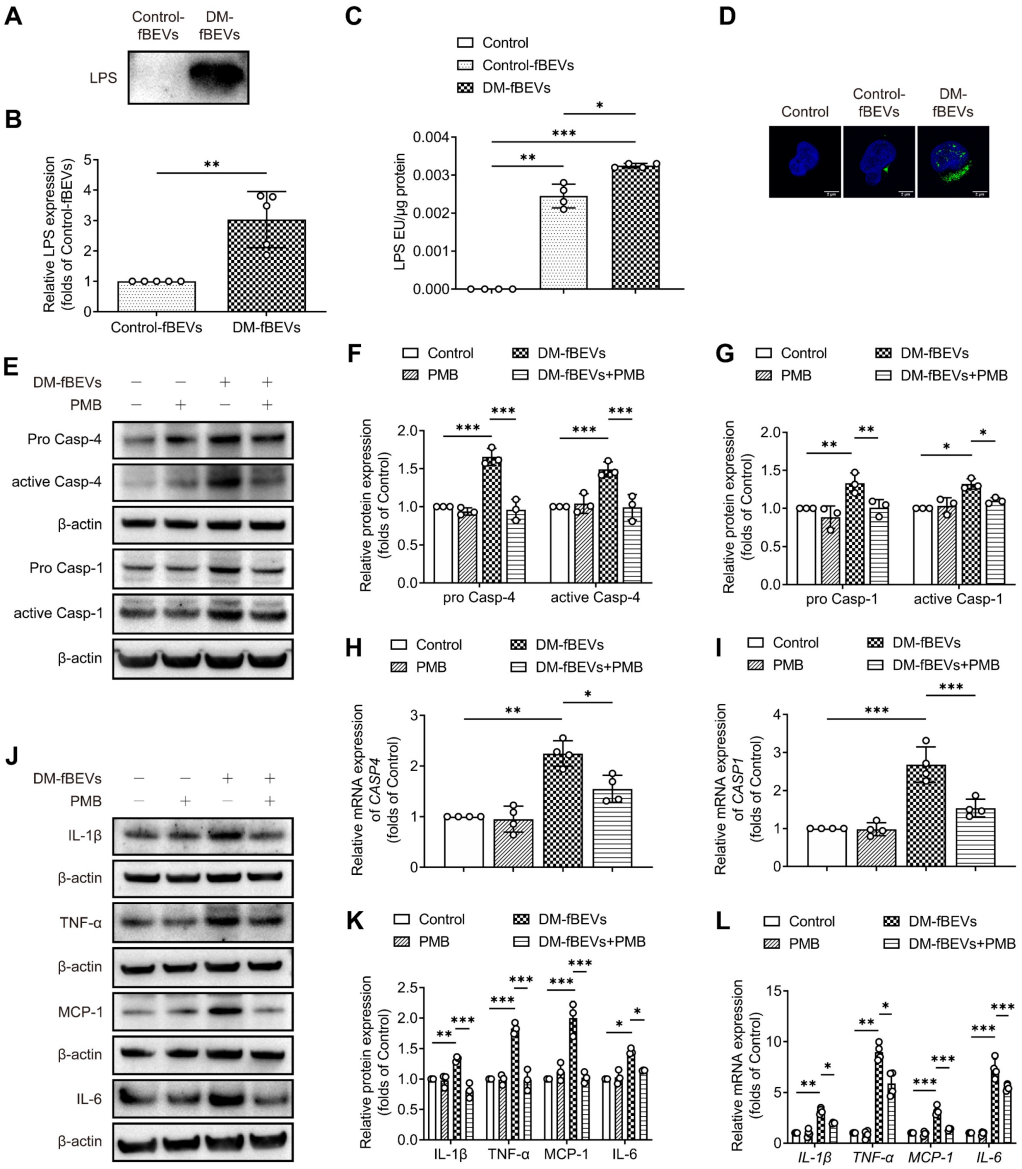
** OMVs-derived LPS contributes to tubulointerstitial inflammation**. **(A-B)** LPS levels of the fBEVs lysates with the same protein level from different groups were measured by Western blot analysis of lipid A (n=5). HK-2 cells were treated without (Control) or with 5 μg/mL Control-fBEVs or DM-fBEVs for 24 h. **(C)** LPS levels in the cytosolic fractions of HK-2 cells were measured by the LAL assay (n=4). **(D)** LPS in HK-2 cells was visualized with an anti-lipid A antibody, shown in confocal laser scanning microscopy images (scale bar, 2 μm, original magnification × 1000). HK-2 cells were treated without (Control) or with 30 μg/mL PMB, 5 μg/mL DM-fBEVs or DM-fBEVs pretreated with PMB (DM-fBEVs+PMB) to neutralize LPS. **(E-G)** Protein expression levels of pro caspase-4, active caspase-4, pro caspase-1 and active caspase-1 were measured by Western blot analysis (n=3). **(H-I)** mRNA expression levels of *CASP4* and *CASP1* were measured by qRT-PCR analysis (n=4). **(J-K)** Protein expression levels of IL-1β, TNF-α, MCP-1 and IL-6 were measured by Western blot analysis (n=3). **(L)** mRNA expression levels of *IL-1β*, *TNF-α*, *MCP-1* and *IL-6* were measured by qRT-PCR analysis (n=4). *P < 0.05; **P < 0.01; ***P < 0.001.

OMVs are filled with components from Gram-negative bacteria, among which LPS is abundant. Given the efficient uptake of OMVs by TECs, we investigated whether OMVs could deliver LPS to HK-2 cells, targeting the caspase-11 pathway. To characterize the effect of OMVs-derived LPS, fBEVs lysates were immunoblotted with an antibody to lipid A, indicating the LPS content in vesicles. As shown, the LPS level of DM-fBEVs lysates was much higher than that of Control-fBEVs under the same protein level (Figure 9A-B). We also extracted cytosol from HK-2 cells treated with Control-fBEVs or DM-fBEVs and assessed LPS levels using the LAL assay. The results showed that the LPS level in DM-fBEVs-treated cells was notably increased (Figure 9C), which was also reflected by the immunofluorescence staining results (Figure 9D). Together these findings revealed that the level of LPS was higher in fBEVs from diabetic rats. In addition, we sought to determine whether abundant LPS could be associated with the effects of fBEVs on caspase-11 pathway and inflammation activation in HK-2 cells. PMB was used to counteract LPS in fBEVs. The results showed that the effect of DM-fBEVs on the caspase-11/caspase-1 pathway activation was inhibited after pretreatment with PMB (Figure 9E-I), along with improved inflammatory response (Figure 9J-L). These results indicate that LPS is partly responsible for the activation of the caspase-11/ caspase-1 pathway and subsequent inflammatory response induced by OMVs from the DM group.

## Discussion

There is a growing awareness that inflammation-mediated tubulopathy is predictive of the progression of DKD 28, 29. However, the driving factors have not been fully elucidated. It has been reported that gut microbiota dysbiosis plays key roles in metabolic inflammation 30, 31. Therefore, we sought to investigate the effects of gut microbiota on tubulointerstitial inflammation and injury in DKD. This study preliminarily found that OMVs derived from gut microbiota participated in the interaction between imbalanced microbiota and TECs through the delivery of LPS and regulation of the cellular inflammatory response, thereby contributing to tubular epithelium injury and progression of DKD.

Abnormal gut microbiota has been reported in DKD. Winther *et al.* demonstrated that gut microbiota and plasma metabolites in DKD patients were different depending on the albuminuria levels 32. Our previous study also revealed that in STZ-induced diabetic rats, the bacterial abundance and diversity decreased, while acetate-producing bacteria increased 6. Additionally, some interventions can blunt the disease progression by improving dysbiosis. High-fiber diet increased the expansion of short chain fatty acid (SCFA)-producing bacteria with less podocyte injury and interstitial fibrosis. SCFA treatment decreased the expression of inflammatory and fibrotic genes in diabetic kidneys and in cultured TECs and podocytes stimulated with high glucose 3.

Administration of bacterial polysaccharides modulated the microbiota and alleviated the proinflammatory reaction in DKD mice 33, 34. Probiotic supplementation exerted beneficial effects on glucose and lipid homeostasis, oxidative stress and inflammation in diabetic hemodialysis patients 35. In this study, we observed that both depletion and transplantation of microbiota downregulated serum levels of cytokines and their expressions in tubulointerstitium of diabetic rats, along with improved tissue injuries. These data further revealed that gut microbiota influenced the tubulointerstitial inflammation pathway and pathophysiological consequence of DKD, likely through some kinds of mediators.

OMVs are byproducts of Gram-negative bacteria and emerging studies have focused on their *in vivo* distribution and roles in different diseases. Jiang *et al.* reported that OMVs spread to liver, lung, spleen and kidney at 3 h after intraperitoneal injection and induced systemic and lung inflammation 36. Luo *et al.* reported that fBEVs from mice fed with a high-fat diet, administered via tail vein or jejunum injection, translocated to metabolic tissues, such as epididymal fat and skeletal muscles, leading to tissue inflammation and insulin resistance 20. Lee *et al.* reported that orally administrated *Paenalcaligenes hominis*-EVs caused colitis and penetrated the brain barrier through the blood and vagus nerve, thus contributing to cognitive impairment 21. *In vitro*, OMVs could be internalized by cells, such as human microvascular endothelium cells, HeLa cells and human gingival fibroblasts 37, 38. Since OMVs can cross-talk with diverse mammalian cells and modulate the host immune response 18, we investigated the involvement of OMVs in low-grade tubule inflammation. Remarkably, fBEVs extraction, evaluation and quantification confirmed the increase of the amount of OMVs in the diabetic gut, suggesting that OMVs could be a potential etiological factor in DKD. We found that the gut-vascular barrier was disrupted and more OMVs accumulated in tubular cells in diabetic rats. To obtain direct information on the interaction between OMVs and TECs, we exposed HK-2 cells to fBEVs. The results revealed that OMVs could be internalized by HK-2 cells by various endocytic pathways, thereby promoting a subsequent inflammatory response. Moreover, DM-fBEVs administered to mice by oral gavage led to the accumulation of OMVs in TECs and induced systemic and local inflammation and tubulointerstitial injuries. To the best of our knowledge, these findings provide the first direct evidence for the association between OMVs and tubulointerstitial inflammation in DKD progression.

We also explored the potential mechanism by which OMVs induced tubular inflammation. It has been reported that OMVs can activate different signaling pathways in target cells. It was shown that OMVs could trigger the activation of the toll like receptor 4 (TLR4) or nucleotide-binding oligomerization domain-containing protein-dependent nuclear factor kappa-light-chain-enhancer of activated B cells pathway 37, 38. Losier *et al.* found that pathogen-derived OMVs promoted the activation of adenosine monophosphate activated protein kinase and inhibition of mechanistic target of rapamycin complex 1, thereby inducing xenophagy in multiple cell lines 39. Deo *et al.* demonstrated that OMVs from pathogenic microbes induced mitochondrial dysfunction in microphages by activating the Bcl-2 antagonist killer-caspase 3/7 pathway 40. In recent years, increasing evidence suggests a critical role of caspase-11 pathway in regulating kidney cellular homeostasis and the development of DKD. Upregulation of caspase-11 induced tubule cell damage and IL-18 release in cisplatin or ischemia-reperfusion-induced acute kidney disease 41. Caspase-11 was found to directly interact with caspase-1 in TECs, leading to maturation of IL-1β and renal fibrosis in unilateral ureteral obstruction mice 42. Caspase-11 was also associated with inflammation and injury of podocytes in diabetic mice 23. Our experiments revealed that the expressions of caspase-11 and caspase-1 were upregulated in the kidney of diabetic rats. Oral administration of DM-fBEVs activated the caspase-11/caspase-1 pathway and inflammatory reaction in kidney and promoted tubulointerstitial injury. Similar results were also found in HK-2 cells directly exposed to DM-fBEVs. In contrast, inhibition of OMVs by microbiota depletion and fecal transplantation inhibited caspase-11/caspase-1 activation and tubular abnormalities. In addition, knockdown of caspase-4 *in vitro* downregulated caspase-1 and the inflammatory response. Thus, the caspase-11 pathway mediated the OMVs-induced tubulointerstitial inflammation and renal injury.

OMVs contain various cargoes, including proteins, DNA, RNA, lipids and virulent factors, which transfer bacterial information to target cells 17, 18. LPS, recognized as an endotoxin from gut, has been reported to be associated with DKD. A clinical study showed that higher serum LPS activity was correlated with the progression of kidney disease in diabetic patients 43. LPS administration *in vivo* or *in vitro* was found to induce renal cell apoptosis 44, 45. In addition, LPS is also shed in OMVs and modulates host cells 17. It has been demonstrated that *Escherichia coli*-derived OMVs induced more severe cytotoxicity, intracellular caspase-11 and LPS interaction, and inflammation in peritoneal macrophages compared with a single LPS treatment 46, 47. Oral gavage of *Paenalcaligenes hominis*-EVs more potently impaired cognitive functions than orally administered LPS 21. We found that LPS richly present in DM-fBEVs could be delivered into HK-2 cells. DM-fBEVs-induced caspase-11/caspase-1 activation and inflammation were inhibited by the LPS neutralizer PMB, suggesting that LPS is a major effector derived from DM-fBEVs that triggered inflammation in tubular cells. It was reported that extracellular LPS within *Escherichia coli*-OMVs activated TLR4 on the cell surface of bone marrow-derived macrophages, consequently inducing guanylate binding proteins (GBPs) expression via TIR domain-containing adaptor-inducing interferon-β/interferon regulatory factor/type I interferons pathway. Then GBPs mediated caspase-11 activation, possibly through promoting LPS release from endocytosed OMVs or directly connecting LPS on the membrane of OMVs with caspase-11 25, 48. Intracellularly, lipid A component of LPS could bind directly with caspase-activation and recruitment domain of caspase-11 with high affinity and then promote its oligomerization and activation through autoproteolytic cleavage, in TLR4-independent manner 49-51. Nonetheless, further study is needed to explore the mechanism of the LPS release from OMVs into cytosol and subsequent interaction between LPS and caspase-11.

## Conclusions

This study demonstrated that increased OMVs contributed to tubulointerstitial inflammation and renal injury in the early stage of DKD with the involvement of LPS released from OMVs. The caspase-11/caspase-1 pathway activation was found to be involved in the potential mechanism by which tubulointerstitial inflammation was modulated by OMVs. Our findings pinpoint TECs as target cells of bacterial OMVs and provide new insight into the relevance of dysbiosis and the pathophysiology of DKD.

## Supplementary Material

Supplementary figures and table.

## Abbreviations

DKD: diabetic kidney disease
OMVs: outer membrane vesicles
DM: diabetes mellitus
IL: interleukin
CKD: chronic kidney disease
TECs: tubular epithelial cells
EVs: extracellular vesicles
LPS: lipopolysaccharide
STZ: streptozotocin
AB: antibiotics
FMT: fecal microbiota transplantation
PBS: phosphate-buffered saline
BCA: bicinchoninic acid
TEM: transmission electron microscopy
NTA: nanoparticle tracking analysis
PAS: periodic acid Schiff' s
DAPI: 4, 6-diamidino-2-phenylindole
TNF-α: tumor necrosis factor-α
MCP-1: monocyte chemoattractant protein-1
AQP-1: aquaporin 1
LTL: lotus tetragonolobus lectin
HK-2: human kidney-2
siRNA: small interfering RNA
PMB: polymyxin B
LAL: limulus amebocyte lysate
ELISA: enzyme linked immunosorbent assay
HRP: horse radish peroxidase
NAG: N-acetyl-β-D-glucosaminidase
qRT-PCR: quantitative real-time polymerase chain reaction
ompF: outer membrane protein F
ZO-1: zona occludens 1
fBEVs: fecal bacterial extracellular vesicles
NCR: NAG-creatinine ratio
SCFA: short chain fatty acid
TLR4: toll like receptor 4
GBPs: guanylate binding proteins
