# Supplementary material for: Outer membrane vesicles derived from gut microbiota mediate tubulointerstitial inflammation: a potential new mechanism for diabetic kidney disease
Source: Theranostics. 2023 Jul 9;13(12):3988–4003. doi: 10.7150/thno.84650 (PMC10405836; doi:10.7150/thno.84650)
Supplement: Supplementary file 1 — Supplementary figures and table. [file thnov13p3988s1.pdf]

Supplementary Materials

Figure S1

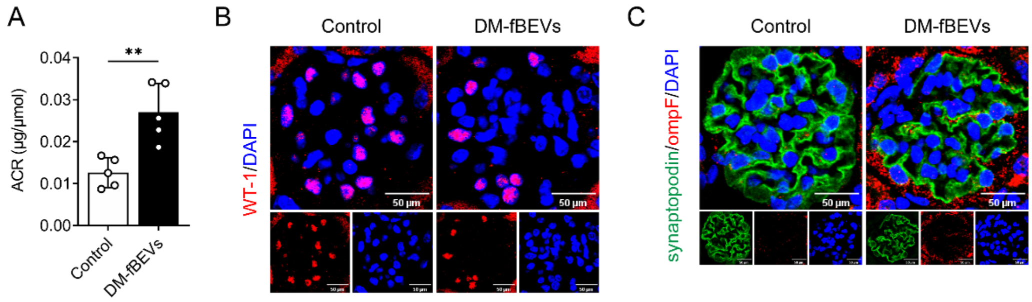

Figure S1. Orally administrated DM-fBEVs translocate to glomerulus and induce podocyte damage. (A) Urinary albumin/ creatinine ratio (ACR) was measured in mice (n=5). $^{**}P < 0.01$. (B) WT-1^{+} (red) podocytes were stained by immunofluorescence staining. Nuclei were stained with DAPI (blue) (scale bar, 50 μm, original magnification × 400). (C) The presence of ompF^{+} OMVs (red dots) in mouse podocytes marked with synaptopodin (green) was determined by immunofluorescent staining. Nuclei were stained with DAPI (blue) (scale bar, 50 μm, original magnification × 400).

Figure S2

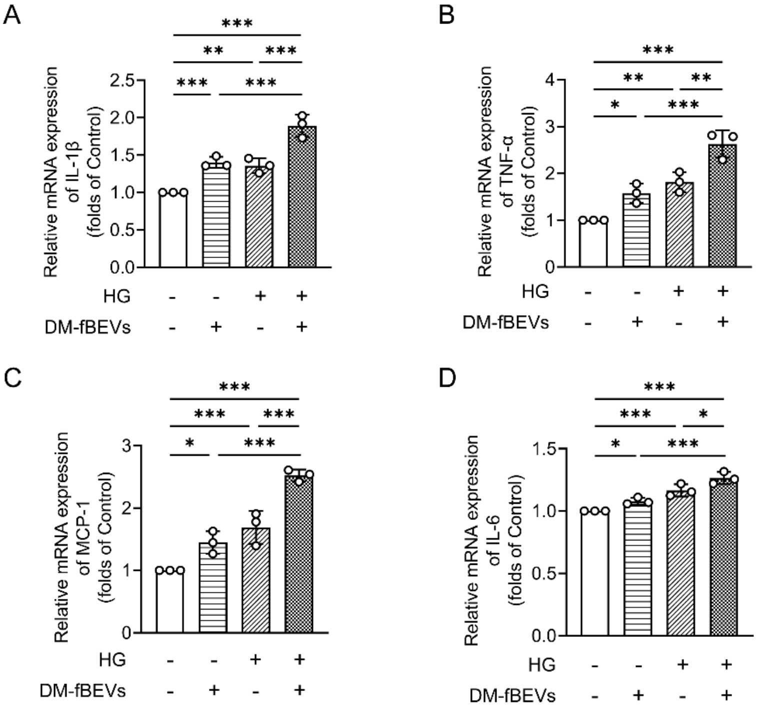

Figure S2. HK-2 cells were treated with high glucose (HG) (30 mmol/L), DM-fBEVs (5 μg/mL) and HG (30 mmol/L) together with DM-fBEVs (5 μg/mL) for 24 h. mRNA levels (n=3) of *IL-1β*, *TNF-α*, *MCP-1* and *IL-6* were measured by qRT-PCR analysis. **P* < 0.05; ***P* < 0.01; ****P* < 0.001.

Figure S3

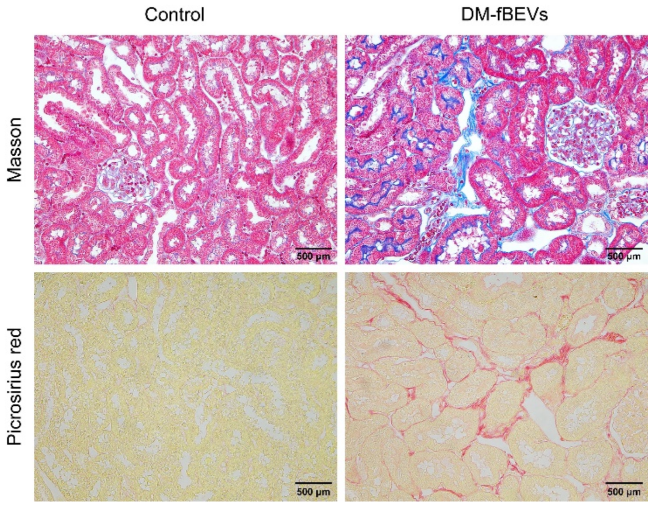

Figure S3. Tubulointerstitial pathological changes were detected by Masson staining and Picrosirius red staining. (Scale bar, 500 μm, original magnification × 400).

Figure S4

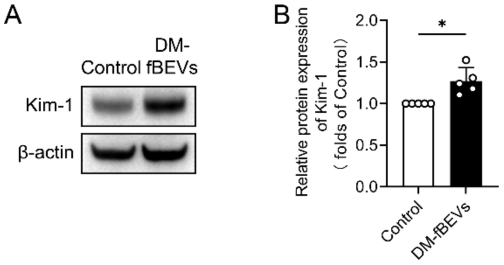

Figure S4. Protein expression levels of Kim-1 in the renal cortex of Control and DM-fBEVs gavage mice were detected by Western blot analysis (n=5). *$P < 0.05$.

Table S1. Primers for qRT-PCR

Gene	Forwards (5'-3')	Reverse (5'-3')
Homo CASP4	TCGGAAGGTACAGCAATCAT	TGCCAGGAAAGAGGTAGAAA
Homo CASP1	ACAGACAAGGGTGCTGAACAA	TCGGAATAACGGAGTCAATCA
Homo IL-1β	ATGATGGCTTATTACAGTGGCAA	GTCGGAGATTCGTAGCTGGA
Homo TNF-α	GGAAAGGACACCATGAGC	CCACGATCAGGAAGGAGA
Homo MCP-1	TGAAGCTCGCACTCTCG	GTGACTGGGGCATTGATT
Homo IL-6	ACTCACCTCTTCAGAACGAATTG	CCATCTTTGGAAGGTTCAGGTTG
Homo GAPDH	GGTGTGAACCATGAGAAGTATGA	GAGTCCTTCCACGATACCAAAG
